# Rat lungworm (*Angiostrongylus cantonensis*) active larval emergence from deceased bubble pond snails (*Bullastra lessoni*) into water

**DOI:** 10.1017/S0031182023000434

**Published:** 2023-07

**Authors:** Phoebe Rivory, Rogan Lee, Jan Šlapeta

**Affiliations:** 1 Sydney School of Veterinary Science, Faculty of Science, The University of Sydney, Camperdown, NSW 2006, Australia; 2 NSW Health Pathology, Centre for Infectious Diseases and Microbiology Lab Services, Level 3 ICPMR, Westmead Hospital, Westmead, NSW 2145, Australia; 3 Westmead Clinical School, Faculty of Medicine and Health, The University of Sydney, Sydney, Australia; 4 Sydney Infectious Diseases Institute, The University of Sydney, NSW 2006, Australia

**Keywords:** angiostrongyliasis, *Angiostrongylus cantonensis*, aquatic snail, *Bullastra lessoni*, experimental infection, intermediate host, rat lungworm, transmission pathways

## Abstract

*Angiostrongylus cantonensis* (the rat lungworm) is a zoonotic parasite of non-permissive accidental (dogs, humans, horses, marsupials, birds) hosts. The 3^rd^ stage larvae (L3s) in the intermediate host (molluscs) act as the source of infection for accidental hosts through ingestion. Larvae can spontaneously emerge from dead gastropods (slugs and snails) in water, which are experimentally infective to rats. We sought to identify the time when infective *A. cantonensis* larvae can autonomously leave dead experimentally infected *Bullastra lessoni* snails. The proportion of *A. cantonensis* larvae that emerge from crushed and submerged *B. lessoni* is higher in snails 62 days post-infection (DPI) (30.3%). The total larval burden of snails increases at 91 DPI, indicating that emerged larvae subsequently get recycled by the population. There appears to be a window of opportunity between 1 and 3 months for infective larvae to autonomously escape dead snails. From a human and veterinary medicine viewpoint, the mode of infection needs to be considered; whether that be through ingestion of an infected gastropod, or *via* drinking water contaminated with escaped larvae.

## Graphical abstract

**Figure:**
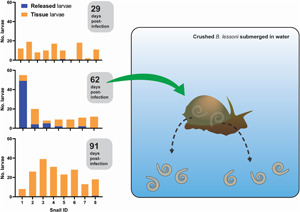

## Introduction

Host-finding behaviours used by a range of parasites, whether they be active (in response to cues) or passive, dictates transmission success by increasing the chances of encountering and establishing infection in a host (Esch *et al*., 2014). An example of a parasite that uses passive transmission is *Angiostrongylus cantonensis* (rat lungworm, the causative agent of potentially fatal neural angiostrongyliasis). This parasitic worm utilizes a broad range of intermediate molluscan hosts as a vehicle for ingestion and subsequent life cycle completion in the definitive *Rattus* spp. host (Cowie, 2013). It has been noted that *A. cantonensis* has an active mode of transmission – by migrating out of dead/dying gastropods into water, where 3^rd^ stage larvae (L3s) remain infective to rats (Cheng and Alicata, 1964; Richards and Merritt, 1967; Crook *et al*., 1971). Consequently, emergent L3s of *A. cantonensis* are considered a tangible route of infection for humans and other accidental hosts (Wallace and Rosen, 1969; Alto, 2001; Howe *et al*., 2019).

In early studies on the life history of *A. cantonensis*, the reported time taken for 1^st^ stage larvae (L1s, which are shed in rat feces) to develop into infective L3s in the intermediate gastropod host is 19–25 days (Bhaibulaya, 1975; Cross, 1979). Larvae manually liberated *via* digestion from gastropod tissue 3 weeks ( = 21 days) after infection with *A. cantonensis* were infective to rats (Wang *et al*., 2012). Recent experiments investigating the active migration of *A. cantonensis* L3s out of dead/dying gastropods when drowned are scarce, and in both cases the snails and slugs were at least 37 days post-infection (DPI) before submersion experiments were undertaken (Modrý *et al*., 2020; Pai *et al*., 2022). Early studies on emerged larvae's infectivity to rats either used naturally infected gastropods or did not disclose how long the gastropod was infected prior to passage (Cheng and Alicata, 1964; Richards and Merritt, 1967; Crook *et al*., 1971). It remains unclear at what timepoint post-infection the *A. cantonensis* L3s are able to actively migrate from dead/dying gastropods into water. This behavioural strategy used by *A. cantonensis* may have substantial implications for the seasonality and epidemiology of neural angiostrongyliasis, by exploiting multiple transmission routes.

The aim of this study was to determine a timepoint post-infection when the *A. cantonensis* larvae are able to escape drowned dead gastropods, a feature of the *A. cantonensis* life cycle which is biologically important for transmission. We sought to investigate the proportion of *A. cantonensis* larvae that spontaneously emerge from experimentally infected, crushed bubble pond snails (*Bullastra lessoni*) submerged in water; and whether the time since initial exposure (29, 62 and 91 DPI) impacted this active larval migration.

## Materials and methods

### Angiostrongylus cantonensis

The *A. cantonensis* isolate (SYD.1) used in this study was originally isolated from a wild black rat (*Rattus rattus*) captured in Mosman, Sydney, Australia in 1997 – as cited in Červená *et al*. (2019). The life cycle of this strain has since been maintained in laboratory-reared bubble pond snails (*Bullastra lessoni*) and Wistar rats (*Rattus norvegicus*) at Westmead Hospital, Sydney, Australia [Western Sydney Local Health District (WSLHD) Animal Ethics Committee approval number: 8003.03.18]. The L1s used for the following experiments were harvested a day in advance *via* the Baermann technique from infected rat feces (Mackerras and Sandars, 1955). L1s were then separated from other fecal material immediately before use by 2 rounds of gentle centrifugation at 1000 ***g*** for 10 min, and resuspension with reverse-osmosis (RO) water.

### Aquatic snails (*B. lessoni*)

The laboratory-reared native Australian aquatic snail used in this study, the southern bubble pond snail (*B. lessoni*, family Lymnaeidae), was originally isolated from Wyong, NSW, Australia. A population of *Angiostrongylus*-free snails, and a separate tank of long-term infected snails are currently maintained at Westmead Hospital. The infected snail population is regularly maintained by exposing groups of naïve snails (kept separately) to thousands of L1s in a large glass Petri dish, and adding them to the infected tank – as described in Pai *et al*. (2022). Throughout the duration of these experiments, all *B. lessoni* were held in 25°C and 80% humidity aquarium tanks fitted with an air pump, and crushed marble substrate. The snails were offered rinsed lettuce *ad libitum* and the tank was washed regularly to remove dead snails, decaying feed, superfluous eggs and juvenile snails.

### Protocol for the detection of actively emerged and remaining larvae

Whole living snails were individually placed in 15 mL falcon tubes and quickly euthanized by crushing, then 10 mL of deionized water was added and allowed to settle for 24 h at room temperature. For the detection of actively emerging larvae, the water surrounding the snail tissue was siphoned out and centrifuged at 400 ***g*** for 10 min. The supernatant was discarded, and the pellet was resuspended with approximately 1 mL of deionized water and a few drops of iodine solution and placed on a watch glass. *Angiostrongylus* larvae were examined under a Nikon SMZ-2B stereomicroscope (Nikon Corporation, Tokyo, Japan) at a minimum of 3.3× magnification and were counted. The remaining tissue from the previously crushed snail was retained in the original falcon tube and frozen at −18°C for later tissue digestion. For the enumeration of larvae which remained in the snail, the retained tissue was artificially digested in 10 mL of digestion solution (10 g pepsin powder 2000 FIP-U per g, 8.5 g NaCl and 30 mL HCl 37% per litre of distilled water) (Penagos-Tabares *et al*., 2019). Tubes were vortexed briefly, and then placed in a 36°C B.Braun Certomat WR shaking water bath (BBI Biotech, Berlin, Germany) for 24 h. The lysate was poured through a wire mesh sieve to remove remaining detritus and the flow through was centrifuged at 400 ***g*** for 10 min, then resuspended and examined microscopically as above.

### Larval emergence from crushed snails 29, 32, 62 and 91 DPI

**Figure 1.**
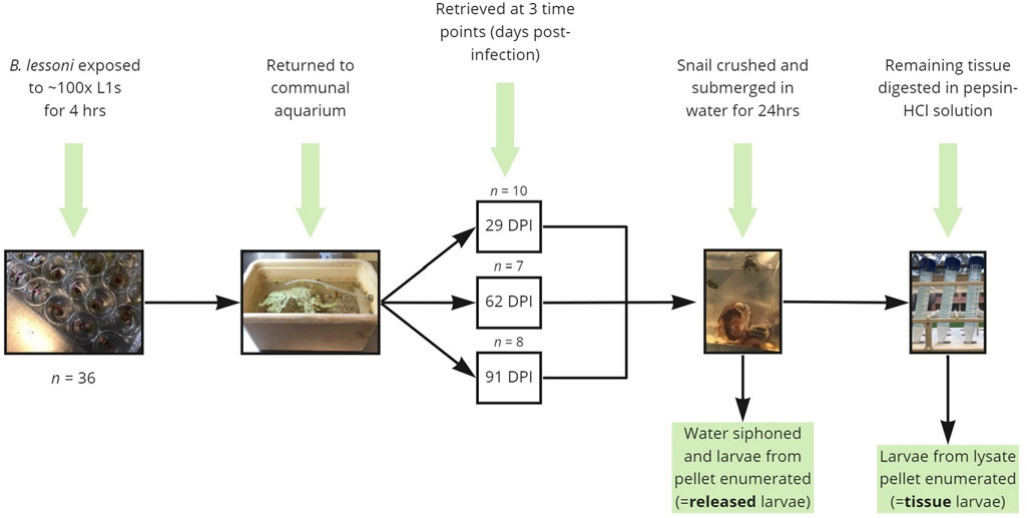
Diagrammatic summary of the experimental protocols used in the current study to determine the proportion of *Angiostrongylus cantonensis* larvae which spontaneously emerge from aquatic snails (*Bullastra lessoni*) at 29, 62 and 91 DPI.

To determine the time post-infection at which larvae will spontaneously emerge from crushed and submerged snails, uninfected *B. lessoni* snails (*n* = 36) were individually placed in each 2 cm well of a cell culture plate with a lid, pre-filled with 1 mL RO water and approximately 100 *A. cantonensis* L1s. After 4 h of exposure at room temperature, snails were removed from the plate and placed in a fresh communal aquarium. At each time interval of 29 (*n* = 10), 62 (*n* = 7) and 91 (*n* = 8) DPI, a subset of snails was removed and transported to The University of Sydney Laboratory for Veterinary Parasitology for submersion and digestion (Fig. 1). Note that over the course of the experiment, snail natural die-off was observed (the population declined by 9 between 1 and 62 DPI, and a further 2 between 62 and 91 DPI). Initial time intervals (29, 62 and 91 DPI) were selected so that counting of emergent larvae would begin after the stated L1–L3 development time (i.e. 25 days) and to accommodate for the expected life span of laboratory snails (3–4 months).

For clarification of unexpected early-stage results (29 DPI), an additional experimental group was added; with naïve snails (*n* = 10) being exposed to approximately 100 L1s and returned to a new communal aquarium before processing at 32 DPI.

### Larval emergence from consistently infected laboratory-maintained snail population

For contextualization and interpretation of the results, positive control snails were processed. Individual snails from the consistently infected laboratory-maintained population (*n* = 10), and from the *Angiostrongylus*-free population (*n* = 2), were selected at random and transported to The University of Sydney Laboratory for Veterinary Parasitology for submersion and digestion utilizing the same protocol.

### Statistical analyses

Raw experimental data were recorded using Microsoft Excel, Microsoft Corporation. All statistics and graphs produced in this paper were created using GraphPad Prism version 9.4.0 for Windows (San Diego, CA, USA). Number of released and tissue larvae for individual snails were visualized using bar charts. Total larval burden was calculated by the sum of emerged and remaining larvae per snail, and visualized for the 29, 62 and 91 DPI groups using a scatterplot. Total larval burden values were tested for normality using the Shapiro–Wilk test. A 1-way analysis of variance (ANOVA) using the Kruskal–Wallis non-parametric test was performed with subsequent Dunn's multiple comparisons. To factor for any snail weight-related variance, the average snail weight (g) was also visualized using a scatterplot and tested for normality (Shapiro–Wilk test) prior to performing a 2-way ANOVA. Statistical level of significance for all tests was set to *α* = 0.05.

## Results

### Larval emergence was highest at 62 DPI and negligible at 29, 32 and 91 DPI

**Figure 2.**
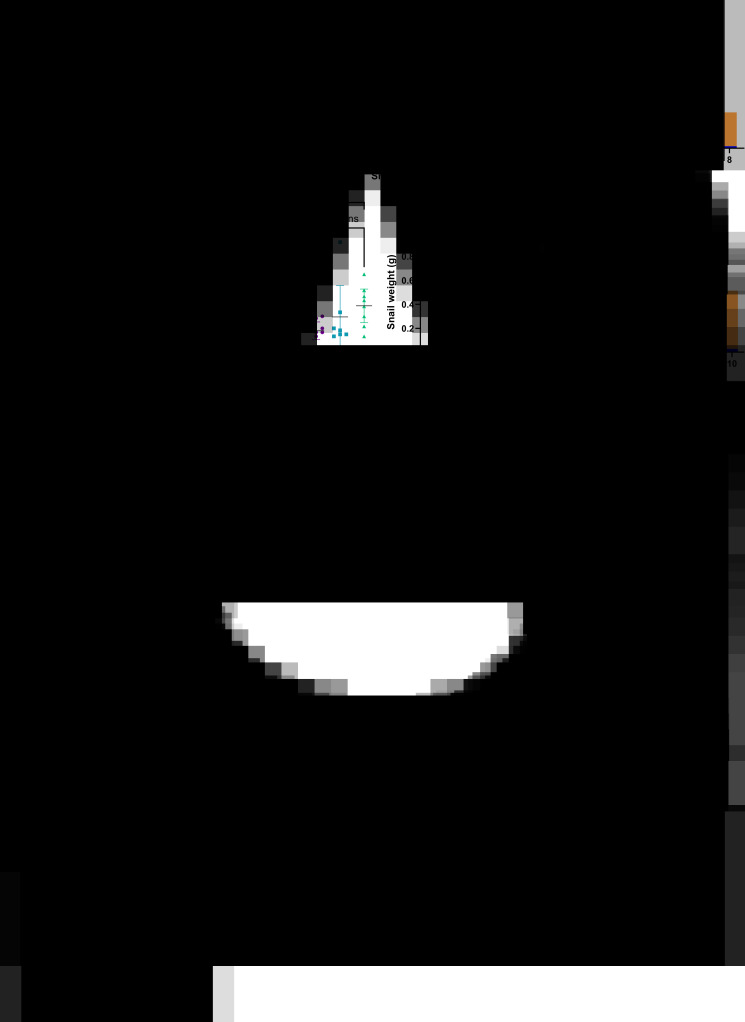
Analyses of *A. cantonensis* larval burden in experimentally infected bubble pond snails (*B. lessoni*). (A–C) Number of *A. cantonensis* larvae per snail which spontaneously migrated out from individual deceased and crushed snails over 24 h (released) *vs* the number which remained in snail tissue (tissue). Co-housed snails were initially exposed to approximately 100 *A. cantonensis* L1s, and retrieved at 3 time points: 29 (A), 62 (B) and 91 (C) DPI. (D) Number of *A. cantonensis* larvae which spontaneously migrated out from individual deceased and crushed snails (*n* = 8) over 24 h (released) *vs* the number which remained in snail tissue (tissue). Snails were initially exposed to approximately 100 *A. cantonensis* L1s and retrieved at 32 DPI. (E) Individual total larval burden of co-housed snails initially exposed to approximately 100 *A. cantonensis* L1s, and retrieved at 29, 62 and 91 DPI. Total larval burden was the sum of larvae per snail which spontaneously emerged and remained in the snail. The mean total larval burden for each group is represented by a black line. The 95% confidence intervals are shown. Statistical significance (*) was calculated using a 1-way ANOVA (Kruskal–Wallis test) with a Dunn's multiple comparisons test. (F) Individual weight (g) of co-housed snails initially exposed to approximately 100 *A. cantonensis* L1s, at the time of retrieval (29, 62 and 91 DPI). The mean weight (g) for each group is represented by a black line and 95% confidence interval is shown. Statistical significance (*) was calculated using a 2-way ANOVA. (G) Number of *A. cantonensis* larvae per long-term infected snail which spontaneously migrated out from individual deceased and crushed snails over 24 h (released) *vs* the number which remained in snail tissue (tissue). Experimental infection and incubation period of the snails in this cohort was not controlled, as they were from the laboratory-maintained long-term positive population.

We exposed 36 snails to ~100 freshly harvested 1^st^ stage larvae of *A. cantonensis* SYD.1. At 29 DPI, randomly selected snails (*n* = 10) were euthanized. Larvae that emerged in water ( = ‘released’ larvae), and larvae remaining in the tissue liberated *via* digestion ( = ‘tissue’ larvae) were counted. Only a single snail (10%, 1/10) released a single larva of *A. cantonensis* at 29 DPI, and all snails (10/10) were positive for tissue larvae (Fig. 2A). At 62 DPI, 5 (71%, 5/7) randomly selected snails released larvae of *A*. *cantonensis* into the water (Fig. 2B). The number of released *A. cantonensis* larvae ranged from 0 to 49, with a mean of 8.9 at 62 DPI. The percentage of larvae released compared to the sum of all counted larvae ranged 0–89.1% (mean 30.3%) across the 62 DPI snails. All snails in this group (*n* = 7) were positive for tissue larvae. At 91 DPI, only 1 snail (12.5%, 1/8) released *A. cantonensis* in water, shedding only a single larva. All snails (8/8) were positive for remaining tissue *A. cantonensis* larvae at 91 DPI (Fig. 2C).

To confirm the inability of larvae to be released at 1 month post-infection, we repeated the experiment with 10 *A. cantonensis*-infected snails. At 32 DPI, only 1 snail (1/8) was positive for released *A. cantonensis* larvae (single emerged larva detected) (Fig. 2D). All snails (8/8) were positive for tissue larvae.

### Total larval burden of *A. cantonensis* larvae increased in snails 3 months post-infection

The total larval burden of individual snails ranged from 1 to 19 larvae for the 29 DPI group, from 8 to 55 larvae for the 62 DPI group and from 8 to 39 larvae for the 91 DPI group. The calculated means for each group were 10.8 (29 DPI), 17.7 (62 DPI) and 23.3 (91 DPI) (Fig. 2E).

The 29 and 91 DPI groups passed the Shapiro–Wilk normality test (*P* > 0.05); however, the 62 DPI group did not pass (*P* < 0.05). A 1-way ANOVA using the Kruskal–Wallis non-parametric test was performed to account for the non-normal data distribution. The day post-infection was a significant source of variation (*P* < 0.05), so a Dunn's multiple comparisons test was performed. There was a significant difference between the means of the 29 and 91 DPI groups (*P* < 0.05).

### Snail weight had no significant relationship over the 3 months

Recorded snail weight (g) at the time of euthanasia ranged from 0.24 to 0.78 for the 29 DPI group, from 0.48 to 0.76 for the 62 DPI group and from 0.57 to 0.71 for the 91 DPI group. The calculated means for each group were 0.52 (29 DPI), 0.61 (62 DPI) and 0.62 (91 DPI) (Fig. 2F). All 3 groups passed the Shapiro–Wilk normality test, so a 2-way ANOVA was performed. There was no significant difference between the weight of snails euthanized at 29, 62 and 91 DPI (*P* > 0.05).

### Consistently infected laboratory-maintained snail cohort

Of the 10 randomly selected consistently infected laboratory-maintained snails, 9 (90%, 9/10) were positive for *A. cantonensis* larvae released into water (Fig. 2G). The number of released larvae per snail ranged from 0 to 35 (mean 7.8). The percentage of larvae released compared to the sum of all counted larvae ranged from 5.2 to 50% (mean 30.2%). Nine snails (90%, 9/10) were positive for *A. cantonensis* larvae in the tissue, with a range of 0–73 larvae per snail (mean 26.6). The total larval burden in this cohort ranged from 0 to 104 larvae, with a mean of 34.4 larvae per snail. One individual (snail no. 1) apparently harboured no larvae. Naïve negative control snails (*n* = 2) were negative for both released and tissue larvae.

## Discussion

The route of transmission of *A. cantonensis* for their accidental hosts such as humans and dogs remains debated (Prociv *et al*., 2000). While the ingestion of intermediate hosts (molluscs) is the traditionally cited route of transmission, infection initiated by the *A. cantonensis* larvae released from dead or drowned molluscs is anecdotally reported and may be more common than previously considered (Wallace and Rosen, 1969; Alto, 2001; Howe *et al*., 2019; Johnston *et al*., 2019). Morphologically, the development of *A. cantonensis* L3s takes 19–25 days in the susceptible intermediate host (Bhaibulaya, 1975; Cross, 1979) and these larvae are infectious to its definitive rat host (Cheng and Alicata, 1964; Richards and Merritt, 1967; Crook *et al*., 1971). Here, we show that larvae in an intermediate host (bubble pond snails, *B. lessoni*) were not readily able to escape the dead intermediate host for at least 32 DPI, thus exceeding the traditional maximum of 25 days for development of infectious *A. cantonensis* L3s reported by Bhaibulaya (1975). Depending on the yet unknown magnitude of the risk of released *A. cantonensis* L3s in disease emergence in humans, dogs and wildlife, the demonstrated timing needs to be considered with angiostrongyliasis seasonality. In Sydney, Australia, a case–control study by Walker *et al*. (2015) demonstrated that canine neural angiostrongyliasis (CNA) has a seasonal peak in May, with some evidence of spatial clustering. Considering the 9–14 day delay from ingestion of the parasite to onset of clinical signs in CNA cases (Jindrak and Alicata, 1970), and high terrestrial snail activity around after warm, wet weather, typically seen in autumn (March–May) (Stanisic *et al*., 2010) we cannot disregard the plausibility that larvae released into water 1 month post-infection of the gastropod could contribute to the observed CNA seasonality.

The concept of intermediesis, *sensu* Colella *et al*. (2015), describes the transmission of infective larval stages between intermediate hosts – which has since been demonstrated with *A. cantonensis* L3s from dead *Veronicella sloanii* (pancake slug) and *Lissachatina fulica* (giant African snail) being infective to *Pomacea maculata* (spotted apple snail) by Modrý *et al*. (2020). This mechanism may increase the number of infected intermediate hosts available to the definitive hosts, allowing for the parasite's persistence in the environment – ultimately assisting its spread (Colella *et al*., 2015). Despite regular cleaning of aquariums and removal of deceased snails in the present study, it appears that *B. lessoni* snails beyond 62 DPI had accumulated larval loads. This phenomenon may be a result of (1) infective L3s actively migrating out of living snails, or being passively released by dead snails which subsequently get recycled by new conspecific hosts (intermediesis); or (2) residual L1s on the surface of snails failing to penetrate the snail at the time of exposure and acting as free circulating L1s (as snails were not washed prior to returning to the aquarium). Although, we predict the total larval burden would have increased by 32 DPI if the latter was the primary mechanism. This host-switching strategy could help sustain infective *A. cantonensis* L3s in aquatic populations by allowing for longer survival time, where the exploitation of an intermediate host is essential for its survival.

Experimental and natural infections in slugs and snails show remarkable variation in larval burden and development of *A. cantonensis* larvae in tissues both within and between various species (Richards and Merritt, 1967; Wallace and Rosen, 1969; Tesana *et al*., 2009; Qvarnstrom *et al*., 2013; Kim *et al*., 2014). Snails' susceptibility to infection and level of exposure likely depends on its' habitat and a variety of behavioural and physiological factors (Ash, 1976; Kim *et al*., 2014). In the current study, the observed total larval burden in individual snails showed extreme volatility (ranging from 1 to 55 across all snails initially exposed to ~100 L1s, and from 0 to 104 in long-term infected snails). This may be partly due to the difficulty controlling the exact number of L1s penetrating and/or being consumed by the snail (Pai *et al*., 2022). It does suggest, however, that there is large intraspecific phenotypic variation for tolerance to the parasite. Nevertheless, our results further corroborate the understanding that the epidemiology of this parasite in the intermediate host is largely stochastic, and dependent on the composition of the local gastropod community in which it cycles.

Although ingestion of (part or whole) infected gastropods is accepted as the main transmission route for accidental hosts, we confirm that larvae can naturally migrate from dead gastropods into water, which therefore can act as a natural source of infection. The risk of infection *via* this route is likely higher between 1 and 3 months post-infection of the gastropod (*B. lessoni*, in this instance). The potential for other gastropod species to release larvae spontaneously when drowned, and the optimal time post-infection for this phenomenon to occur undoubtedly requires investigation. Recycling of infective larvae within aquatic gastropod communities *via* intermediesis may increase the chances for L3s to infect rats (for life cycle completion) and accidental hosts. The increased larval emergence from crushed and submerged *B. lessoni* raises considerations for transmission dynamics and seasonality of infection for natural, intermediate and paratenic hosts.

## Data Availability

Data generated in this study are available at: https://dx.doi.org/10.25833/nz39-ks60.
